# The effect of seaweed enriched bread on carbohydrate digestion and the release of glucose from food

**DOI:** 10.1016/j.jff.2021.104747

**Published:** 2021-12

**Authors:** Matthew D. Wilcox, Paul Cherry, Peter I. Chater, Xing Yang, Moaz Zulali, Edward J. Okello, Chris J. Seal, Jeffrey P. Pearson

**Affiliations:** a Biosciences Institute, Newcastle University, Framlington Place, Newcastle upon Tyne NE2 4HH, UK; b Population Health Sciences Institute, Faculty of Medical Sciences, Newcastle University, Newcastle NE2 4HH, UK; c College of Science, Engineering and Food Science, University College Cork, T12 K8AF Cork, Ireland^1^

**Keywords:** *Ascophyllum nodosum*, Carbohydrate Digestion, Glucose, Polyphenol, Seaweed

## Abstract

Two seaweeds; *Ascophyllum nodosum* and *Fucus vesiculosus*, were incorporated into bread at 0.5 and 2% and their effect on blood glucose *in vivo* and carbohydrate digestion *in vitro* were studied.

In the five way randomised placebo controlled double blind pilot trial (n = 10) each volunteer consumed 100 g of available carbohydrate (from bread) and their blood glucose was measured over two hours.

The breads were tested in a human digestion model and compared against control bread and control bread with the equivalent amount of seaweed.

In the pilot human study the enriched breads did not cause any significant reductions in iAUC of blood glucose with average reductions of 0.1 ± 44.4%, 8.2 ± 19.3%, 1.0 ± 54.3% and 2.7 ± 31.9% for 0.5% *F.vesiculosus*, 0.5% *A.nodosum*, 2% *F.vesiculosus*, and 2% *A.nodosum* respectively.

However, seaweed added alongside the control bread *in vitro* significantly reduced the level of carbohydrate digestion compared to the control bread.

*F.vesiculosus* or *A.nodosum* can reduce carbohydrate digestion, however baking into bread reduces the effect.

## Highlights

• Seaweeds can reduce carbohydrate digestion in a physiologically relevant model.
• Baking seaweed into bread can reduce its ability to inhibit carbohydrate digestion *in vitro*.
• Adding seaweed alongside bread retains the ability to reduce carbohydrate digestion *in vitro*.

## 1 Introduction

Polyphenols have been widely studied for numerous potential health benefits; helping treat or prevent neurodegenerative diseases by acting through the Nrf2–EpRE pathway (Buendia et al., 2016), inflammatory bowel disease acting as anti-inflammatory agents (Vezza et al., 2016), and metabolic disease including type two diabetes (Umeno et al., 2016).

Brown seaweeds are rich in polyphenols, typically between 0.1 and 1.5% (dry weight) (Schiener et al., 2015, Tabassum et al., 2016), but with *Ascophyllum nodosum* containing higher amounts (6% dry weight) (Parys et al., 2009, Connan, et al., 2004) and *F. vesiculosus* higher again (up to 20% dry weight) (Ovchinnikov et al., 2020).

The major polyphenols found in brown seaweeds are phlorotannins, which are oligomers of phloroglucinol. Phlorotannins are a cell wall component but play a number of roles within seaweed such as protection against ultraviolet radiation damage (Abdala-Diaz, 2014), deterring some herbivores from feeding on them and separately may also have a function in reproduction (Schoenwaelder, 2002).

One potential treatment to aid glycaemic control has used seaweeds and their polyphenols (Murugan et al., 2015), showing promising results *in vitro* (Lordan, 2013) and with *Ecklonia kurome Okamura* in genetically diabetic mice (Xu et al., 2012). Seaweed polyphenols, in general, have been shown to inhibit certain digestive enzymes, for example glucosidases (Xiao, 2013) such as alpha amylase (Pantidos, 2014, Xiao, 2013, Xu et al., 2016) and have ultimately been shown to affect the digestion of carbohydrates and the absorption of sugar (Williamson, 2013). Targeting the action of glucosidases has been shown to be a useful method to help with glycaemic control (Kim et al., 2016).

Although seaweeds may not be common in the western diet, seaweeds have been included into food products. For example, seaweed has been used to reduce the salt content of frankfurters (Jiménez-Colmenero et al., 2010), beef patties (López-López et al., 2011), and black pudding (Fellendorf et al., 2016), it has been included into bread to study its effects on inflammation (Allsopp et al., 2016), as well as cholesterol and blood glucose levels (Hall et al., 2012).

However, the optimum dose and species of seaweed to include in a standard western diet, whilst maintaining its potential for health benefits has not been fully elucidated.

It was hypothesised that the addition of *Ascophyllum nodosum* or *Fucus vesiculosus* to standard white bread will reduce post prandial blood glucose levels compared to control bread alone. Any potential mechanism of reducing blood glucose will also be investigated using an *in vitro* model gut system (MGS).

## 2 Materials and methods

### 2.1 Materials

All reagents were purchased from Sigma with the exception of pepsin (Affimetrix, High Wycombe, UK), gastric like lipase (Amano Enzyme Inc, Nishiki, Japan) and porcine bile was collected fresh from a local abattoir. The flour, salt, sugar, oil, and yeast were all purchased from Asda Stores Ltd (Leeds, UK). Whole blood glucose was measured using an On-Call ® EZ blood glucose monitoring system, compromising of On-Call® Plus Blood Glucose Test Strips, and On-Call® EZ Blood Glucose Meter (measurement range: 1.1–33.3 mmol/L; ACO1N Laboratories Inc., San Diego, USA). *Ascophyllum nodosum* and *Fucus vesiculosus* were a gift from LEHVOSS Gee Lawson (Congleton, UK). The seaweeds had a polyphenol content of 11.5 ± 4.7 mg gallic acid equivalents (GAE) /g of seaweed extracted in deionised water for *Ascophyllum nodosum* and 165.9 ± 59.3 mgGAE/g for *Fucus vesiculosus*. Using the method of Zhang et al the 2% *Fucus vesiculosus* enriched bread contained 38 mg GAE per 1 g seaweed and the 2% *Ascophyllum nodosum* enriched bread contained 37 mg GAE per 1 g seaweed after baking. The *Ascophyllum nodosum* seaweed before baking into bread contained 62 mg GAE per 1 g, which is in line with Zhang et al’s own findings of 52.6 mg GAE per 1 g (Zhang et al., 2006).

### 2.2 Sample preparation

#### 2.2.1 Test foods

Breads were made in single batches following a standard recipe (Table 1) in NU Food’s pilot kitchen (Newcastle University, UK) by the research team. After baking, the breads were portioned and double wrapped in sealed polythene bags and frozen until required. The bread samples were defrosted at room temperature before use.

**Table 1:** The composition of bread (g/100 g of bread).

	Standard White Bread	0.5% Seaweed Bread	2% Seaweed Bread
Flour	61.4	60.9	59.4
salt	1.3	1.3	1.3
sugar	0.4	0.4	0.4
oil	0.5	0.5	0.5
yeast	0.4	0.4	0.4
water	36.0	36.0	36.0
seaweed	0.0	0.5	2.0
Total	100.0	100.0	100.0
Total Carbohydrate	41.4	41.0	40.1

The dry ingredients (including seaweed when necessary) were added to a Hobart HSM 30 mixer (Hobart UK, Peterborough, UK) and mixed/kneaded using an ED Hook agitator for 10 min at setting 2, the water and oil was added after one minute of the mixing. The dough was incubated at 25 °C for two hours, mixed again for 5 min, placed into bread tins, and then incubated for a further one hour. A Bonnet Equator oven (Hobart UK, Peterborough, UK) was used to bake the bread at 200 °C for 30 min.

During the cooking process the loaves lost on average 11.6% weight (as water), therefore total water content in the cooked bread was 24.4%.

#### 2.2.2 Model gut solutions

The composition of the model gut solutions are described in detail by Houghton et al and are briefly described below (Houghton et al., 2014).

##### 2.2.2.1 Artificial saliva

Artificial saliva was composed of 62 mM sodium di-hydrogen phosphate, 6 mM di-potassium hydrogen phosphate, 15 mM sodium chloride, 6.4 mM potassium chloride and 3 mM calcium chloride and α-amylase from hog pancreas (150 U/L). The pH was adjusted to 7.4.

##### 2.2.2.2 Artificial gastric juice

Artificial gastric juice was composed of 49.6 mM sodium chloride, 9.4 mM potassium chloride, 2 mM potassium di-hydrogen phosphate, 5 mM urea, 500 mg/L pepsin and 400 U/L gastric like lipase.

##### 2.2.2.3 Artificial pancreatic juice

Artificial pancreatic juice was composed of 110 mM sodium bicarbonate, 2.5 mM di-potassium hydrogen phosphate, 54.9 mM sodium chloride, 1 mM calcium chloride, 1.67 mM urea and pancreatin (8x USP) 70 g/L. The solution was filtered through glass wool to remove insoluble material before use.

### 2.3 Methods

#### 2.3.1 Effect on post prandial blood glucose by seaweed enriched breads protocol

Ten healthy males and healthy, non-lactating, non-pregnant females of 18 years or older, not taking prescription drugs and not diabetic or pre-diabetic were recruited by posters and word of mouth from Newcastle University staff and their associates (median 22 years old, range 19–29, 3 females). All volunteers who were enrolled on the study were recruited, randomised and completed all five conditions.

Volunteers received 100 g of available carbohydrate (total carbohydrate minus fibre content) from one of five study breads, in random order. 100 g of available carbohydrate was chosen to achieve the desired dose of seaweed to volunteers whilst maintaining a palatable bread. Volunteers came to the study centre on five separate occasions with at least two days apart to test each of the breads in a random order. The breads were; standard white bread (control), standard white bread containing 0.5% dried *A. nodosum*, standard white bread containing 2% dried *A. nodosum, s*tandard white bread containing 0.5% dried *F. vesiculosus, s*tandard white bread containing 2% dried *F. vesiculosus.*

Volunteers arrived in the morning at the NU-Food research facility after fasting overnight. Fasting blood glucose levels were measured and then volunteers consumed the test bread and drank 250 ml of water within 10 min. Blood was sampled, and glucose levels tested at 15, 30, 45, 60, 90, and 120 min after commencing eating with subjects remaining sitting throughout the sampling period. The procedure was repeated for each of the test breads with at least two days between testing.

The study design was a five way randomised, controlled double blind, trial and was approved by Newcastle University’s Faculty of Science, Agriculture and Engineering Research Ethics Committee for project “Effect of seaweed-enriched bread on postprandial glucose response″. Written, informed consent was obtained from each volunteer prior to their participation in the study.

#### 2.3.2 Carbohydrate digestion analysis in the model gut system protocol

The model gut system was a semi dynamic model that replicates chemical and enzymatic digestion in the mouth, stomach and small intestine and has been described in detail by Houghton et al (Houghton et al., 2014). In brief each experiment contained 5 ml deionised water (with or without sample) and 5 ml synthetic saliva and mixed at 75 rpm for 30 s. This was added to 50 ml synthetic gastric juice (at 37 °C) and the remaining synthetic gastric juice was added at 0.5 ml/min for 60 min and maintained at 37 °C. At 60 min, 25 ml pre-warmed (37 °C) porcine bile was added along with synthetic pancreatic juice which was added at a rate of 0.5 ml/min for 120 min. 1 ml samples were taken at 0, 15, 30, 45, 60 (before and after the addition of porcine bile) 90, 120, 150 and 180 min (the end of the model gut). There were 9 samples tested in the MGS along with their respective controls. The samples were; control bread, 0.5% *A. nodosum* enriched bread, 2% *A. nodosum* enriched bread, 0.5% *F. vesiculosus* enriched bread, 2% *F. vesiculosus* enriched bread, 25 mg *A. nodosum* + control bread, 100 mg *A. nodosum* + control bread, 25 mg *F. vesiculosus* + control bread, 100 mg *F. vesiculosus* + control bread. 5g of bread were added to each run of the model gut system so 25 and 100 mg of seaweed added alongside the control bread are the equivalent to the same weight and amount of seaweed as would be present in the 0.5 and 2% seaweed loaves.

### 2.4 Statistical analysis section

Incremental area under the glucose response curve (AUC) above the fasting glucose concentration was calculated using Prism 6 (GraphPad, La Jolla, CA, USA). Two way ANOVA was used to compare the mean blood glucose level of the human volunteers consuming each of the four test breads and control bread. Data are expressed as mean ± standard error of the mean.

## 3 Results

### 3.1 Effect on post prandial blood glucose by seaweed enriched breads

**Fig. 1:**
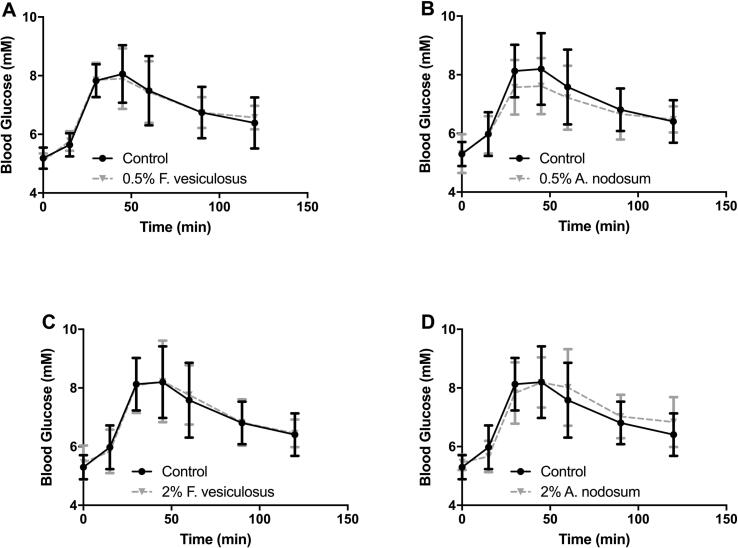
Mean blood glucose concentrations for healthy volunteers. Data for control bread is shown in each of the four panels (●). Panel A – shows the blood glucose concentration after consumption of the control bread (●) and bread enriched with 0.5% *F. vesiculosus* (▾).Panel B – shows the blood glucose concentration after consumption of the control bread (●) and bread enriched with 0.5% *A. nodosum* (▾).Panel C – shows the blood glucose concentration after consumption of the control bread (●) and bread enriched with 2% *F. vesiculosus* (▾).Panel D – shows the blood glucose concentration after consumption of the control bread (●) and bread enriched with 2% *A. nodosum* (▾).

The volunteers consumed each of the breads within the first 10 min of the study, all participants finished the full portion and there were no problems reported with acceptability. There was no statistical difference between either of the four enriched breads compared to the control bread, at any time point (Fig. 1).

Reductions in the mean peak blood glucose concentration for both the 0.5% *F. vesiculosus* (peak of 8.1 ± 0.9 mM) and 0.5% *A. nodosum* (peak of 8.3 ± 0.6 mM) enriched breads compared to control bread (peak of 8.7 ± 1.0 mM). The 2% enriched breads both increased the peak blood glucose (peak of 9.0 ± 0.9 mM) and (peak of 8.8 ± 0.9 mM) for *F. vesiculosus* and *A. nodosum* respectively. However, none of these changes were statistically different to the control.

The differences between the peak blood glucose between the enriched breads and the control bread, when using volunteers as their own control, reduced the peak by 0.5 ± 1.1 mM and 0.4 ± 0.8 mM for 0.5% *F. vesiculosus* and 0.5% *A. nodosum* respectively. The two enriched breads at higher concentrations however, increased the peak with an average increase of 0.3 ± 0.7 mM and 0.1 ± 1.0 mM respectively for *F. vesiculosus* and *A. nodosum*. Although, none of these changes were statistically significant.

All the enriched breads reduced the incremental area under the curve compared to the control bread, by an average of 0.01 ± 60.31, 16.40 ± 35.83, 13.83 ± 89.86, and 3.55 ± 54.15 mol.min/l for 0.5% *F. vesiculosus*, 0.5% *A. nodosum*, 2% *F. vesiculosus*, and 2% *A. nodosum* respectively. This accounted for an average percentage reduction in incremental area under the curve of 0.1 ± 44.4, 8.2 ± 19.3, 1.0 ± 54.3 and 2.7 ± 31.9% for 0.5% *F. vesiculosus*, 0.5% *A. nodosum*, 2% *F. vesiculosus*, and 2% *A. nodosum* respectively. These reductions in actual incremental area under the curve or as a percentage were not statistically significant.

### 3.2 Carbohydrate digestion analysis in the model gut system

**Fig. 2:**
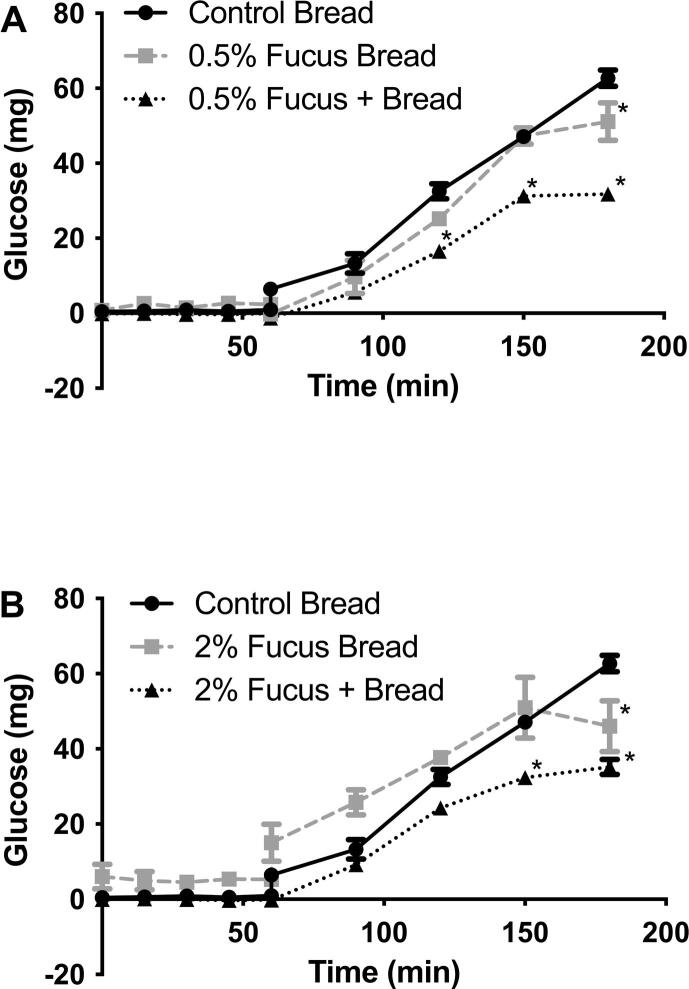
Glucose release in the Model Gut System with the addition of Control bread (with and without *F. vesiculosus*) and *F. vesiculosus* enriched bread. Panel A – shows control bread (●), bread enriched with 0.5% *F. vesiculosus* (■), and control bread with added *F. vesiculosus*, equivalent to the 0.5% enriched bread (▴). Panel B – shows control bread (●), bread enriched with 2% *F. vesiculosus* (■), and control bread with added *F. vesiculosus*, equivalent to the 2% enriched bread (▴).

**Fig. 3:**
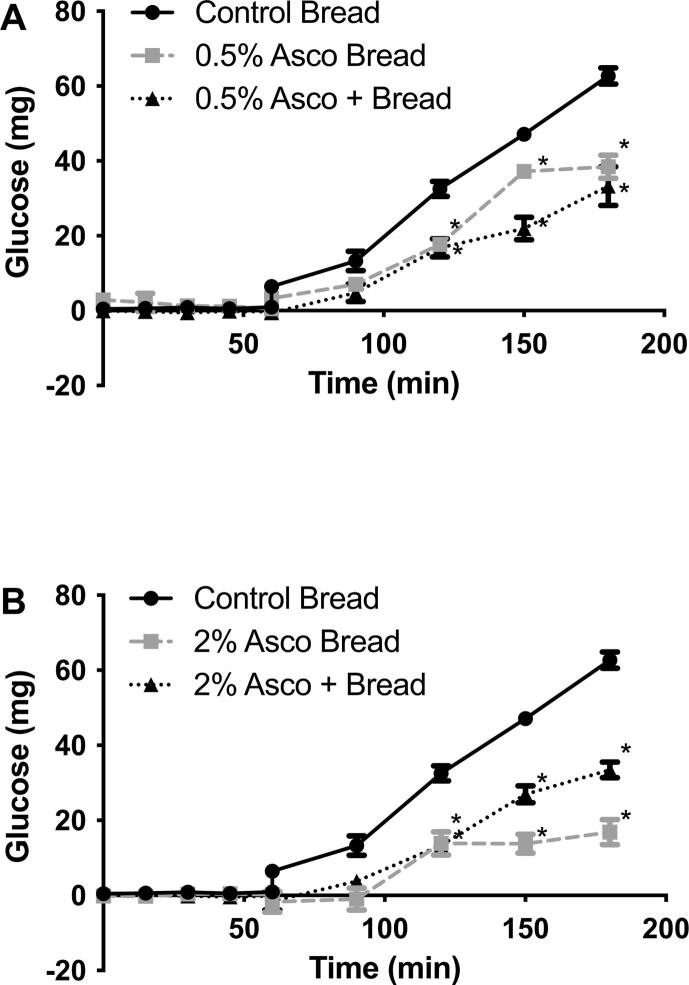
Glucose release in the Model Gut System with the addition of Control bread (with and without *A. nodosum*) and *A. nodosum* enriched bread. Panel A – shows control bread (●), bread enriched with 0.5% *A. nodosum* (■), and control bread with added *A. nodosum*, equivalent to the 0.5% enriched bread (▴). Panel B – shows control bread (●), bread enriched with 2% *A. nodosum* (■), and control bread with added *A. nodosum*, equivalent to the 2% enriched bread (▴).

As expected, there was little or no digestion and release of glucose from any of the breads in the gastric phase of the model gut system. Only after the addition of the carbohydrate digesting enzymes from the pancreas, in the small intestinal phase of digestion, was there significant release of glucose. The control bread releases 31.3 mg/hour glucose during the small intestinal phase of the MGS to a maximum of 62.7 ± 2.2 (SEM) at the end of digestion (Fig. 2, Fig. 3).

There was no significant reduction in AUC for either of the *F. vesiculosus* enriched breads compared to the control bread and only at the final time point was there a significant reduction in glucose release compared to the control for both 0.5% and 2% *F. vesiculosus* enriched breads (Fig. 2). However, when the seaweed was added separately (at equivalent to either 0.5% or 2%) along with the bread there were reduction in AUC for both (2086 ± 85.0 and 2510 ± 44.1 respectively Vs control of 3847 ± 135.8 mg.min). There was also a significant reduction in glucose release at specific time points (90, 120, 150 and 180 min) in the model gut system when adding the equivalent to 0.5% *F. vesiculosus* with the bread and at the final two time points (150 and 180 min) when adding the equivalent to 2% *F. vesiculosus* with the bread.

The addition of *A. nodosum* enriched breads reduce the AUC compared to control bread (3847 ± 135.8 mg.min Vs 2569 ± 118.1 and 1114 ± 84.2 for 0.5% and 2% *A. nodosum* enriched breads respectively) (Fig. 3). The same was also true when the free seaweed was added, at equivalent amounts, with the bread. There was a greater (but not significant) reduction in AUC with the equivalent amount of 0.5% *A. nodosum* added compared to the enriched bread (1817 ± 148.7 Vs 2569 ± 118.1 mg.min respectively). Compared to the control bread there was also a significant reduction in AUC when the equivalent amounts of 2% *A. nodosum* were included, this was lower (but not significantly) than the enriched bread at the same amount (1833 ± 129.7 Vs 1114 ± 184.2 mg.min respectively). After the initial 30 min in the small intestinal phase of the model gut system, the addition of *A. nodosum* as either enriched bread or free seaweed, at either amount, significantly reduced the amount of glucose release compared to the control at all subsequent time points.

## 4 Discussion

The inhibitory properties of brown seaweeds and their extracts on digestive enzymes have been shown previously and reviewed elsewhere (Chater et al., 2015). Polyphenol extracts of brown seaweeds have been shown to be particularly effective against the activity of carbohydrate digesting enzymes (Roy et al., 2011, Nwosu et al., 2011, Murugan et al., 2015, Lordan, 2013, Xu et al., 2012), as well as in this study when unbaked seaweed has been tested in the MGS, however there is only limited *in vivo* data.

All the seaweed enriched bread tested in human in this study did show a reduction in incremental area under the blood glucose curve, however the reductions were not significantly different to the control bread. This study was not powered to find significance, but difference of this size would require greater than 250 volunteers even for the group with the greatest difference. However, due to the small sample size (n = 10) and measurement variability this calculation may also vary with more data.

A previous study investigating the effect of seaweed enriched bread on subsequent energy intake, also measured post-prandial blood glucose levels and also found no difference in response between the seaweed enriched bread and the control bread (Hall et al., 2012). That study also used *A. nodosum,* the same species of seaweed used in one arm of the current study with volunteers consuming the same total amount of seaweed as the highest concentration used in this study (4 g seaweed per serving). The *A. nodosum* seaweeds when tested alone was shown to have an inhibitory effect on carbohydrate digestion *in vitro* both in this study and previously (Goni et al., 2002), and a reduction in insulin response *in vivo* (Paradis et al., 2011). However, in this study as with Hall *et al* (Hall et al., 2012) little difference in post prandial blood glucose response was seen, potentially indicating that during the bread baking process, the bioactive(s) responsible for inhibiting carbohydrate digestion are reduced, destroyed or not bioavailable.

The reduction in efficacy of *F. vesiculosus* and low concentration *A. nodosum* seaweed once baked into bread could be due to polyphenol degradation. As some polyphenols are known to be heat labile, for example, the total polyphenol content of heated (180 °C) olive oil reduced with time (to 35–52%) (Roodaki, 2016). Similarly, the temperature the seaweed is dried at can affect the total polyphenol content. Increasing the drying temperate of *F. vesiculosus* from 35 °C to 75 °C reduce the polyphenol content by nearly half (greater than46%) (Moreira et al., 2016). So, it is plausible that the heat from the baking process of the bread sufficiently reduces the polyphenol content to a level that becomes ineffective for inhibition of carbohydrate digestion. However, hot water extraction of polyphenols from *F. vesiculosus* and *A. nodosum* have been shown to be effective inhibitors of carbohydrate digesting enzymes *in vitro,* and *in vivo* in rats (Roy et al., 2011). Although the exact extraction process was not reported, it would be appropriate to assume greater than 35 °C water would have been used in the process. Although not statistically different, the highest concentration *A. nodosum* enriched bread inhibit carbohydrate digestion to a greater extent than that of the free seaweed. It may be possible that *A. nodosum* polyphenols are less susceptible to heat degradation.

The interactions of polyphenols and various proteins are well known and has been proposed as a potential mechanism for the inhibition of the carbohydrate digesting enzymes (Bandyopadhyay et al., 2012). This could potentially be an alternative or complementary theory to the heat degradation of polyphenols as to why there was little inhibition of carbohydrate digestion with the enriched bread. The protein network within bread plays an important role in the structure of the bread and how it rises. It may be possible that during the bread making process with the relatively long incubation (proofing) times (3 h), that the release of water soluble polyphenols could occur and bind with the developing protein network. Resveratrol, a polyphenol commonly found in grapes, has been shown to interact strongly with gliadin, a component of the gluten network (Qiu et al., 2017). Resveratrol was hypothesised to make hydrophobic interactions with multiple gliadin compounds (Qiu et al., 2017). The multiple interactions that resveratrol makes with gliadin may not affect the overall gluten network as they do not block interactions with other gliadin compounds. This would correlate with the lack of observed differences between the heights or volumes of the loaves with and without seaweed. Although not potentially affecting the gluten network, the polyphenols bound to gliadin would not be bioavailable to interact with the carbohydrate digesting enzymes. However, polyphenol availability was not assessed in the model gut system which may have indicated a reduction of free polyphenols when incorporated into bread. However, a loss of polyphenols was observed during the baking process.

Seaweeds that are higher in polyphenol content have a have been shown to have lower level of protein content (Tibbetts et al., 2016). Red seaweeds for example generally have lower polyphenol content than brown seaweeds but a higher level of digestible protein. There are differences between species of the same colour classifications, with *A. nodosum* having significantly lower levels (6.5%) of digestible protein than *F. vesiculosus* (9.6%), both being brown seaweeds (Tibbetts et al., 2016). This inverse relationship between polyphenol content and digestible protein, may demonstrate that, in order to retain the functionality of the polyphenols the potential interaction with proteins need to be kept to a minimum (Tibbetts et al., 2016).

Polyphenols have been shown *in vitro* to be a potent inhibitors of carbohydrate digestion (Lordan, 2013), however, polyphenols are not the only compound found within brown seaweed that can inhibit carbohydrate digestion. Fuciodan extracted from both *A. nodosum* and *F. vesiculosus* has been shown to have an inhibitory effect on alpha amylase and alpha glucosidase (Kim et al., 2014). Traditionally fucoidans were extracted under strong acid conditions to avoid the extraction of alginic acid but are now generally extracted using hot water, as was the fuciodan used by *Kim et al*, with extraction under heat (85 °C) and then with ethanol and concentrated via lyophilisation (Kim et al., 2014). Although the extraction process is unlikely to be repeated under physiological conditions it may be possible that some fucoidan could be bioavailable when seaweed is consumed. Highest concentrations of fucoidan are found in *F. vesiculosus* (9.8 wt%) compared to *A. nodosum* (8.0 wt%) (Fletcher et al., 2017). This would equate to a total of 390 mg and 320 mg respectively for *F. vesiculosus* and *A. nodosum* in the bread or added alongside the bread, if extracted completely. In either, the human digestive tract or the model gut system, the release of this amount of fucoidan would be below the concentration tested previously (Kim et al., 2014) and would be unlikely to have a significant effect on carbohydrate digestion.

Seaweeds contain a range of potential bioactive compounds but to translate *in vitro* effects to *in vivo* situation, optimisation of the source and vehicle are required. The demonstrated effect of seaweed polyphenols inhibiting carbohydrate digestion *in vitro* were not replicated in this *in vivo* study. The *in vitro* digestion model did show the baking process, whether heat degradation, protein interaction, or other mechanism, reduced the effectiveness of the seaweed modulation of carbohydrate digestion. Protecting the seaweed during the baking process through a form of encapsulation would potentially increase the bioavailability of the bioactives at the site required for effect (Arriola et al., 2019). Increasing the bioavailability of the seaweed polyphenols in the small intestine would increase the modulation of carbohydrate digestion translate from *in vitro* to *in vivo*.

## CRediT authorship contribution statement

**Matthew D. Wilcox:** Conceptualization, Data curation, Formal analysis, Funding acquisition, Writing – original draft. **Paul Cherry:** Data curation. **Peter I. Chater:** Conceptualization, Data curation, Formal analysis, Writing – review & editing. **Xing Yang:** . **Moaz Zulali:** Data curation. **Edward J. Okello:** Conceptualization, Funding acquisition. **Chris J. Seal:** Conceptualization, Funding acquisition. **Jeffrey P. Pearson:** Conceptualization, Formal analysis, Funding acquisition, Writing – review & editing.

## Declaration of Competing Interest

The authors declare that they have no known competing financial interests or personal relationships that could have appeared to influence the work reported in this paper.
